# Spectral and Image Integrated Analysis of Hyperspectral Data for Waxy Corn Seed Variety Classification

**DOI:** 10.3390/s150715578

**Published:** 2015-07-01

**Authors:** Xiaoling Yang, Hanmei Hong, Zhaohong You, Fang Cheng

**Affiliations:** College of Biosystems Engineering and Food Science, Zhejiang University, 866 Yuhangtang Road, Hangzhou 310058, China; E-Mails: feeling998@126.com (X.Y.); honghanmei@126.com (H.H.); zijing1979@163.com (Z.Y.)

**Keywords:** waxy corn, hyperspectral imaging, SPA, SVM, PLS-DA, variety classification

## Abstract

The purity of waxy corn seed is a very important index of seed quality. A novel procedure for the classification of corn seed varieties was developed based on the combined spectral, morphological, and texture features extracted from visible and near-infrared (VIS/NIR) hyperspectral images. For the purpose of exploration and comparison, images of both sides of corn kernels (150 kernels of each variety) were captured and analyzed. The raw spectra were preprocessed with Savitzky-Golay (SG) smoothing and derivation. To reduce the dimension of spectral data, the spectral feature vectors were constructed using the successive projections algorithm (SPA). Five morphological features (area, circularity, aspect ratio, roundness, and solidity) and eight texture features (energy, contrast, correlation, entropy, and their standard deviations) were extracted as appearance character from every corn kernel. Support vector machines (SVM) and a partial least squares–discriminant analysis (PLS-DA) model were employed to build the classification models for seed varieties classification based on different groups of features. The results demonstrate that combining spectral and appearance characteristic could obtain better classification results. The recognition accuracy achieved in the SVM model (98.2% and 96.3% for germ side and endosperm side, respectively) was more satisfactory than in the PLS-DA model. This procedure has the potential for use as a new method for seed purity testing.

## 1. Introduction

Waxy corn is the main variety of fresh corn in China. Because of the high content of amylopectin, waxy corn is taken as an important raw material for amylopectin production. To keep the wax and other characteristics, waxy corn must be planted in isolation to prevent being pollinated by corn of other varieties. As a fresh food product, the optimal time of harvest depends on the cultivars. Accordingly, variety classification or identification before planting is very important for waxy corn seeds. Moreover, variety purity is an indispensable criterion for seed quality. Purity is defined as the percentage of expected seeds contained in the tested seed lot. In the process of cultivation, harvesting, storage, and transportation, each production procedure may lead to variety mixing and purity decrease. Purity testing methods vary from each other and can be divided into several types, such as morphology identification, physicochemical or physiochemistry analysis, and molecular identification, *etc.* Most of these methods require professional staff and specialized instruments, and they are often time-consuming. Generally, the variety purity test is completed by trained staff and is based on kernel morphological features like length, shape, and color, *etc.* [1]. Although this method is convenient and economic, its accuracy depends on the experience of the inspectors and is influenced by subjective errors.

Machine vision technology is an alternative method for seed variety classification based on kernel appearance. It can provide objective observation using feature extraction and mathematical modeling. There are literatures reporting that a machine vision system could be applied in classification and variety identification of seeds [2,3,4,5]. Chen *et al.* extracted geometric, shape, and color features (totaling 58 items) of maize seeds. The accuracy reached 88%–100% in classifying five maize varieties with a back propagation neural network (BPNN) classifier [2]. Yan *et al.* extracted color features from the maize crown and the maize side images (including red-green-blue and hue-saturation-value models). Fisher’s discriminant theory was used with the recognition rate of over 93.75% [6]. Manickavasagan *et al.* developed a machine vision system where monochrome images were obtained to differentiate eight wheat classes. Thirty-two textural features were extracted from the gray-scale images. The classification accuracies of linear and quadratic discriminant analysis were among 73%–100% [4]. They also identified wheat class using thermal imaging and the classification accuracy reached 64%–95% [5]. Grillo *et al.* analyzed the images of 10 families representative of the Mediterranean vascular flora seeds and found that image technology was reliable for a statistical classifier in seeds [7]. Machine vision has also been applied in seed quality assessment. Mavi carried out a study to determine the relationship between seed coat and seed quality in watermelon [8].

When morphological characteristics and color were similar among species, it was difficult to classify them by a visual method. Several attempts were made using near-infrared spectroscopy (NIRS) to identify seed variety based on the internal compositions of the seed kernel [9,10,11]. Delwiche *et al.* identified waxy wheat from non-waxy cultivars using NIRS. The results of separating waxy from non-waxy wheat were nearly perfect, but the classification results among three neighboring gene non-waxy classes only achieved an accuracy of 60% [11]. Seregely *et al.* distinguished melon genotypes and found that it was possible to distinguish the hybrid watermelon cultivars using NIRS [10]. Many researchers reported that NIRS could be used as non-destructive technology for measuring the chemical composition of single seed [12]. Moisture, protein, oil, starch of wheat, corn, and other seeds were studied by NIRS [13,14,15,16,17].

However, as hybrid seed cultivars have increased, some seeds may have similar appearances and it is hard to differentiate them by image alone. The growing region, climate, and year also affect the spectral information. There are some limitations when building a discriminant model with image or spectral characters separately. Hyperspectral imaging (HSI) technology is a spectroscopic technique integrated with image information, providing both spatial and spectral data. HSI has been widely evaluated by research groups in the quality assessment of agricultural products and foodstuffs. Cogdill *et al.* analyzed the moisture and oil concentration of maize kernels by NIR HSI [18]. They found that this method was more useful in predicting moisture than oil content. In detection of cereal fungal infection, researchers used HSI technology to analyze cereal samples and achieved preferable results [19,20,21]. HSI was also used in analyzing the hardness of maize kernels and the milling quality of soft wheat [22,23].

In the literature about seed variety classification with HSI, spectral features were mostly chosen for mathematical modeling [24,25,26]. Wang *et al.* investigated the use of spectral and image fusion data to discriminate the variety and quality of rice, giving a good classification accuracy of 94.45% [27]. Zhang *et al.* captured images of maize seeds which were put in a glass dish. The discriminant model was built by the spectral and gray-level co-occurrence matrix (GLCM) of the region of interest (ROI) extracted from the seed images. A prediction accuracy of 98.89% was achieved [26]. However, in their experiments, each variety of corn seeds huddled together and they didn’t focus on the variety of the single kernel. If some seeds from different cultivars mixed in these seeds, they might not be discovered. Thus, the index of seed purity would be decreased. As for hybrid maize seed, the seed purity is very important for producer and planter. Although many methods for seed variety classification have been reported, there’s no previous report on the usage of combined spectral and spatial data to classify the variety of a single maize kernel.

A new procedure implemented for maize seed variety classification was developed. A visible and near-infrared HSI technique was used in classifying different varieties of single corn seeds. Integrated spectral and image features were employed to build a discriminant model. The classification ability of single-type of feature and the difference of both sides of corn seeds were discussed.

## 2. Materials and Methods

### 2.1. Hyperspectral Imaging System

**Figure 1:**
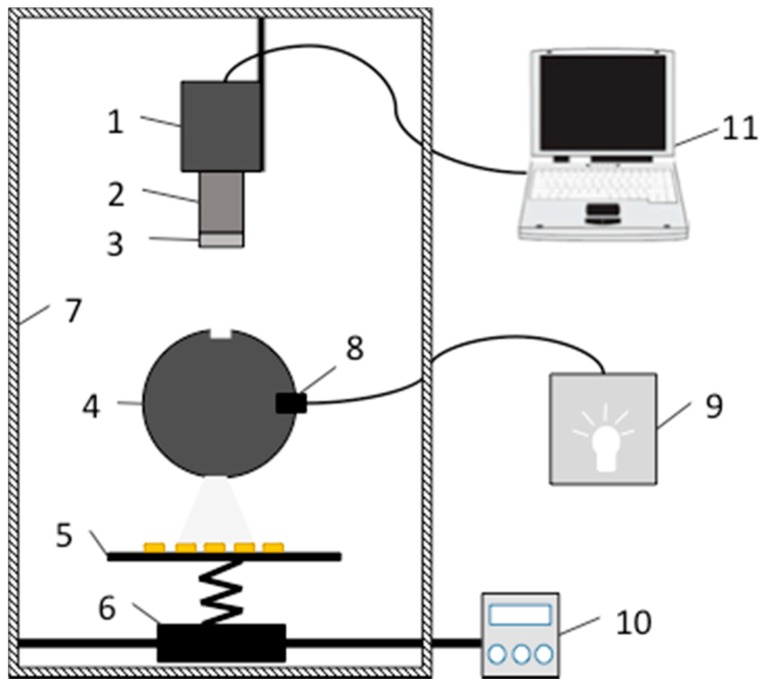
The hyperspectral imaging system: (1) CCD camera; (2) imaging spectrograph; (3) lens; (4) scattering cylinder; (5) sample stage; (6) electrical moving stage; (7) dark room; (8) light source; (9) light source controller; (10) moving stage controller; (11) computer.

The customized visible and near-infrared (VIS/NIR) HSI system was applied. The system consisted of an image spectrograph (Imspector V10E-QE, Spectral Imaging Ltd., Oulu, Finland), a digital charge-coupled device (CCD)camera (C8484-05G, Hamamatsu Photonics, Hamamatsu, Japan), a camera lens (V23-f/2.4 030603, Specim Ltd, Oulu, Finland), illumination and a controller, a sample stage and electric moving stage, a dark room, and a computer (Figure 1). The line scanning image spectrograph had a spectral range of 400–1000 nm and maximum image size of 6.15 × 14.2 mm (spectral × spatial). In this study, the compression mode was set as 2 × 2 binning. The CCD camera has a high-resolution of 1344 (H) × 1024 (V), a wide dynamic range of 12-bit digital output, and high sensitivity in the VIS/NIR region. The illumination consisted of a linear light (P/N 9130, Illumination Technologies, Inc., Elbridge, NY, USA) and a light-scattering cylinder to make the light uniform and even. In order to prevent images from being blurred or deformed, the intensity of illumination, the speed of the electric moving stage, the exposure time of the camera, and the object distance were all set at appropriate values. The object distance was set at 420 mm, the speed of the electric moving stage was fixed at 2.8 mm/s, and the exposure time was set at 90 ms.

### 2.2. Seed Sample Selection and Preparation

**Figure 2:**
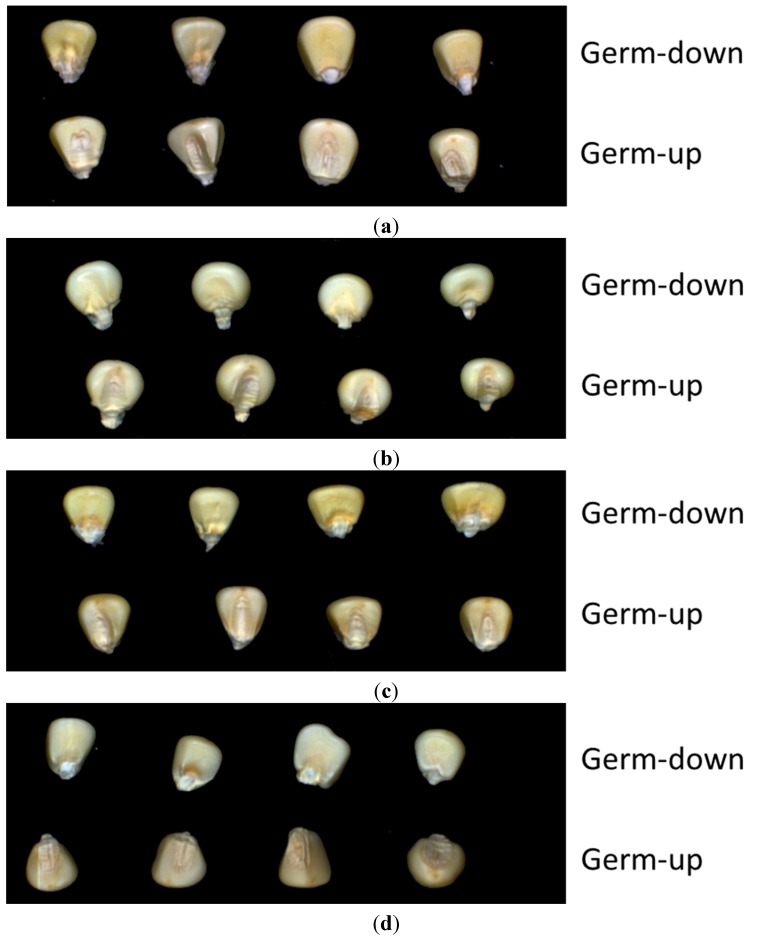
Hyperspectral images of four maize seed varieties: (**a**) HANG; (**b**) SU; (**c**) HU; (**d**) YAN.

The dry seeds of waxy corn cultivars, Hangyunuo No.1 (HANG), Suyunuo 14 (SU), Huyunuo No.1 (HU), and Yanhejin 2000 (YAN) were used for all the experiments in this study. These four white corn cultivars were all hybrid corn and used as fresh corn. The growing periods of these four cultivars varied from 75 days to 85 days. Therefore，their optimal harvest time is various as fresh foods. These seeds were all produced in 2011 in China’s Zhejiang province，thus eliminating the effect of seed age and plant region. After being harvested and dried, the seeds were put in plastic bags and sealed in a plastic box to prevent moisture absorption during store. Before acquisition of the HSI data, the moisture content had been tested to make sure that all the samples had nearly the same moisture content. The final moisture content was 12% before signal acquisition. To explore the feasibility of maize seed cultivar classification using HSI, 150 samples of each variety were selected for imaging. The maize seeds were placed on a black painted platform where HSIs were captured. Considering the imparity of corn seeds, both sides of every seed were explored (Figure 2).

### 2.3. Image Acquisition and Calibration

HSIs of maize seed samples were collected in the wavelength range of 400–1000 nm, including 477 wavebands. The seed samples were put on a black painted sample stage for easier background segmentation during image processing. The images were acquired line-by-line as the samples were passing the view slot of the CCD camera. The reflectance intensities of each pixel of the sample images were recorded at each wavelength slice. The image width was 670 pixels and the height was variable as the set of frames, depending on the number of samples.

Spectral calibration was performed for correction of light source effects using the following formula [24]:

(1) $$I=\frac{I_{0}-B}{W-B\;}$$

where *I* is the relative reflectance intensity of each wavelength slice of HSI, $I_{0}$ is the original reflectance intensity of the hyperspectral image, *B* is the intensity of the dark current, acquired by covering the lens with its cap and turning off the light source, and *W* is the reflectance intensity of the standard white panel (Spectralon, Labsphere Inc., North Sutton, NH, USA). All the corrected images were used in the following spectral information extraction and image processing procedure. The image acquisition and calibration were carried out using Spectracube 2.75b (Spectral Imaging Ltd., Oulu, Finland).

### 2.4. Spectral and Image Feature Extraction

#### 2.4.1. Background Segmentation and Spectra Extraction

**Figure 3:**
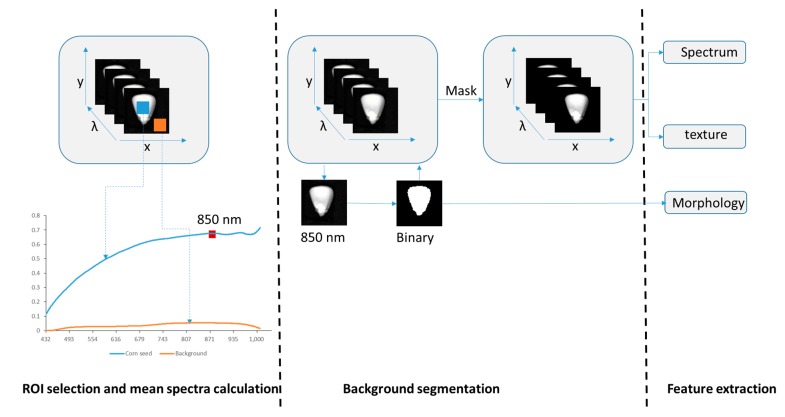
The method of hyperspectral image processing, including ROI selection, background segmentation, and feature extraction.

The image processing procedure, as illustrated in Figure 3, consisted of a series of steps to acquire data and develop the mathematical model. Initially, every image was calibrated with the dark current and white reference image with Equation (1). Successively, the background was removed according to the contrast of relative reflectance intensity between the black background and kernels. Here 20 × 20 pixels were selected from the kernel and background as a region of interest (ROI). Reflected spectra of the two ROIs were averaged and compared. The results show that the highest variance of wavelength between kernels and background is at about 850 nm. Maize kernels were segmented from the images by a threshold process of image at 850 nm to create a mask of the ROIs. Spectra of each pixel from every kernel were extracted and averaged. Background segmentation and spectra extraction were carried out using ENVI 4.8 (ITT Visual Information Solutions, Boulder, CO, USA).

#### 2.4.2. Spectra Preprocessing

**Figure 4:**
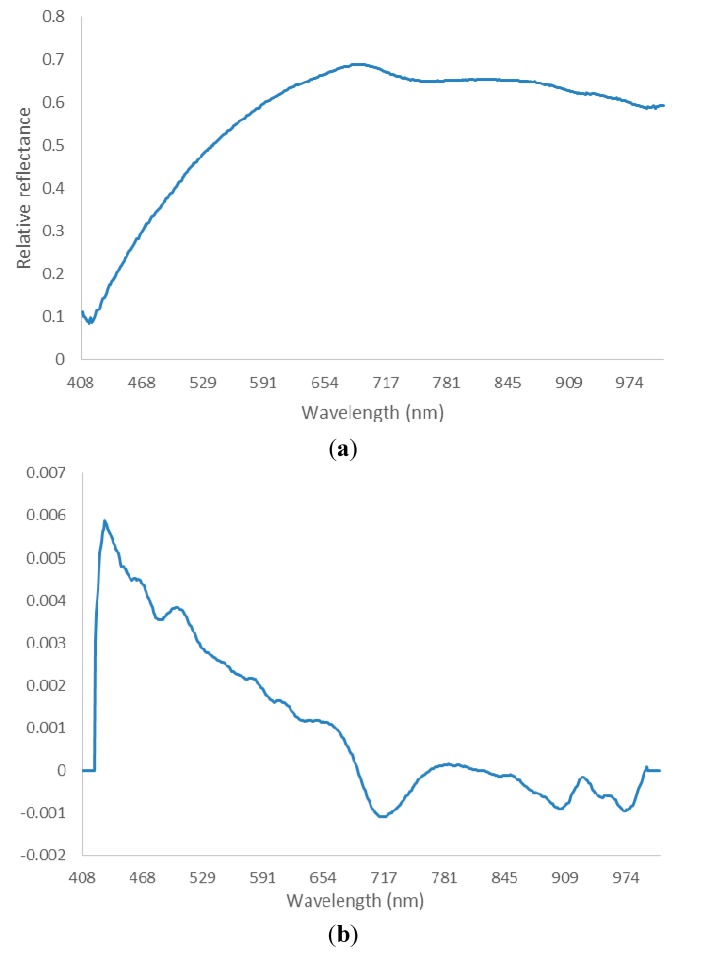
An example of spectra preprocessing. (**a**) Original spectrum; (**b**) spectrum after SG smoothing and derivation.

Data was preprocessed in order to highlight the differences among the study samples. The spectral sensibility of the CCD camera has lower signal-noise ratio near 400 nm and 1000 nm wavelength. Therefore, spectral information from 430 nm to 980 nm was chosen for further analysis. Before selecting optimal wavelength, the spectra were preprocessed by Savitzky-Golay (SG) smoothing filter and derivate. The role of the smoothing filter was to improve signal-noise ratio and eliminate the random noise. The derivate function was used to correct the baseline effects, which could amplify and resolve the overlapped signal. In SG smoothing, the frame size and the polynomial order must be specified. The frame size must be odd and set at 21, and the polynomial order must be less than the frame length and was set at 2 in this experiment. The first derivative was applied on the smoothed spectra by a SG filter. The smoothed and derivate spectra were employed in the following optimal wavelength selection (Figure 4).

#### 2.4.3. Optimal Wavelength Selection

Each of the extracted spectra consisted of 477 bands and suffered from multicollinearity. It was expected that fewer bands could represent the useful information. A small number of variables can reduce the effect of non-related variables and promote the model performance. Successive projections algorithm (SPA) is a forward selection method and is proposed for optimal waveband selection. It can minimize the collinearity among variables with simple operation and has been used in previous research [28,29,30]. SPA was used for dimension reduction and optimal wavelength selection in the data process procedure. The steps of SPA are described below [28]:

Step 0: Assume that the first wavelength k(0) and the number N are given. Let x_j_ = jth column of spectral matrix X_cal_; *j* = 1, 2, …, J.

Step 1: Let S be the reset column set of X_cal_. S doesn’t contain any selected wavelength.

Step 2: Calculate the projection of x_j_ on the subspace orthogonal to x_k(n-1)_ as

(2) $${\text{Px}}_{\text{j}}={\text{x}}_{\text{j}}-({\text{x}}_{\text{j}}^{\text{T}}{\text{x}}_{\text{k}\left(\text{n}-1\right)}){\text{x}}_{\text{k}\left(\text{n}-1\right)}{\left({\text{x}}_{\text{k}(\text{n}-1)}^{\text{T}}{\text{x}}_{\text{k}(\text{n}-1)}\right)}^{-1}$$

Where P is the projection operator.

Step 3: Let k(n) = arg(max$\left\|{\text{Px}}_{\text{j}}\right\|$), j∈S.

Step 4: Let xj = ${\text{ Px}}_{\text{j}}$, j∈S.

Step 5: Let n = n + 1. If n < N, go back to step 1.

End: This time the final selected wavelengths are k(n), n = 0,…, N – 1.

We built the discriminant model using the selected wavelength and calculate the root-mean-square error (RMSE). Then, we changed the number N and did step 1 to step 5 again until the minimal and steady RMSE was acquired. The final selected wavelengths were chosen for following analysis.

Partial least squares (PLS) is another widely used method in wavelength selection for HSI analysis in agriculture and food industry. With the PLS method, variable importance in projection and regression coefficients estimated by PLS can be used to select variables [31]. In our research, the regression coefficients of partial least squares–discriminant analysis (PLS-DA) were used for optimal wavelength selection.

#### 2.4.4. Image Features Extraction

Morphologic features were employed for variety classification in pioneer research [32,33]. According to these previous study results, five morphologic features of each kernel were selected and extracted in the study: area, circularity, aspect ratio, roundness, and solidity. Area is the number of pixels on a segmented single kernel. Circularity is calculated by the Equation (3).

(3) $$circularity=\frac{4\text{π}*area}{perimeter^{2}}$$

Where perimeter is the length of the outside boundary of the selected kernel. The value of circularity indicates a perfect circle when it equals 1. As the value approaches 0, it indicates an increasingly elongated shape. Aspect ratio denotes the ratio of the major axis to the minor axis of fit ellipse for a kernel. Roundness is calculated by the Equation (4).

(4) $$roundness=\frac{4*area}{\pi *major\_axis^{2}}$$

Solidity denotes the ratio of area to convex area. The morphologic features were extracted from the image of 850 nm.

Texture varied among different seed cultivars. In this experiment, we employed the gray-level co-occurrence matrix (GLCM) [34] to describe the texture of maize seed. The fundamental parameters for computing GLCM were defined so that the displacement was set as 1 and the orientation values were 0°, 45°, 90°, and 135°. Energy, contrast, correlation, entropy, and their standard deviations (SD) for all bands of images were calculated. Because of the current equipment limitation, the 1–50 bands were affected by the noise. Instead, we chose 51–477 bands of images to analyze and averaged the extracted GLCM features.

The image and spectral features extraction were applied using Matlab (Version R2012a, The Mathworks Inc., Natick, MA, USA).

### 2.5. Development of Classification Models

Selection of the classifier is the most important factor in the process of building a classification model. There are hundreds of classifiers, and it always confuses researchers to select which is the most appropriate one. For the purpose of finding a classifier that would function well with their dataset, they often tried several of them and then selected the one that had the highest accuracy. Fernández-Delgado evaluated 179 classifiers in order to achieve significant conclusions about the classifier behavior [35]. They used 121 datasets and built classification models. They found that the library for Support Vector Machines (lib-SVM) with the Gaussian kernel and the random forest were the best one. Accordingly, the lib-SVM [36] was employed in our research. C-Support Vector Classification (C-SVC) and radial basis function (RBF) kernel were chosen for multi-classification purpose. For the best performance, the grid-search and five-fold cross-validation were applied to optimize parameters. In order to compare the performance of SVM, the PLS-DA model was developed to classify the seed varieties in this study. The leave-one-out method was applied to the PLS-DA models and the accuracy of every class was calculated.

## 3. Results and Discussion

### 3.1. Spectra of Four Varieties of Maize Seeds

**Figure 5:**
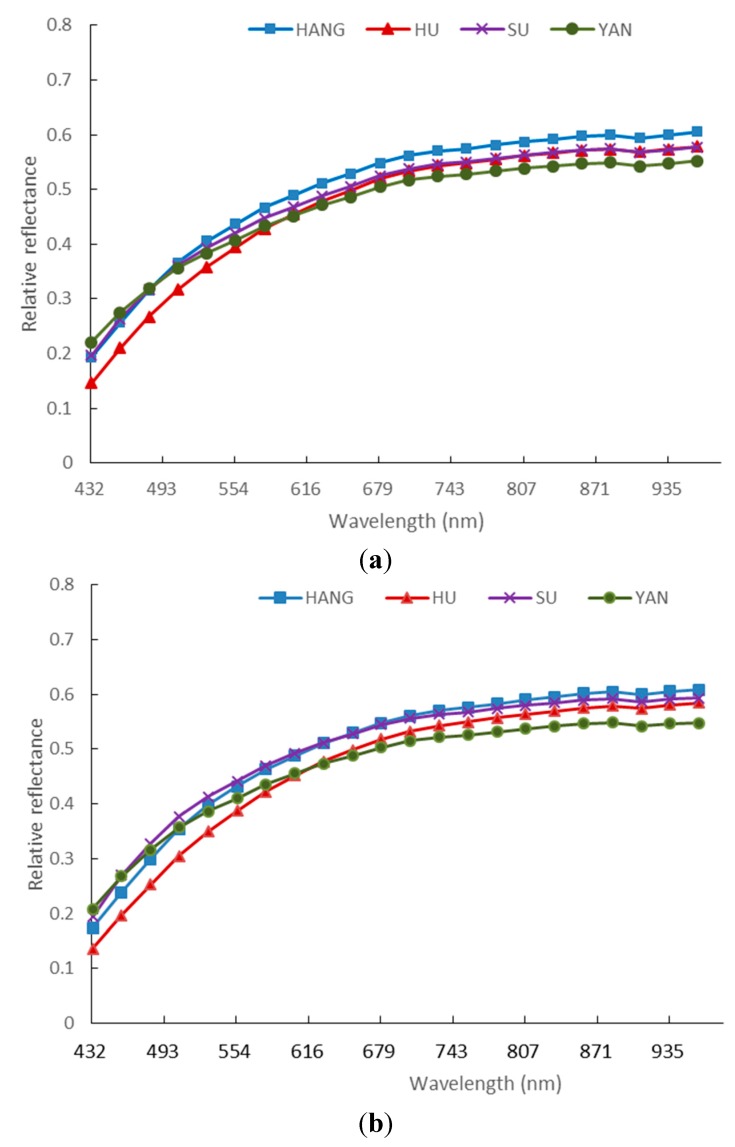
Comparison of spectral reflectance of four maize seed cultivars extracted from germ-up (**a**) and germ-down (**b**) images.

The mean relative reflectance spectra of the four varieties of maize seeds are shown in Figure 5. Comparison results of the four spectra curves showed similar trends between different varieties. In more detail, the germ-up side spectra of HU and SU nearly overlapped at wavelength from 680 nm to 940 nm. However, for germ-down side, the spectra have obvious differences between HU and SU in this region. SU and HANG can be separated at wavelength region from 500 nm to 940 nm in germ-up side images. Meanwhile, SU and HANG also have some differences below 500 nm in germ-down side images. For these four varieties, the wavelength regions that can separate them were inconsistent between the spectra of germ-up and germ-down sides. In Figure 5a, the spectra of HU and SU nearly overlapped in the range of 680–1000 nm. However, in Figure 5b, the spectra of HU and SU were different. These differences may be related to different chromospheres and other components of both sides of maize kernels. For both sides of corn seed, it is necessary to analyze them separately.

### 3.2. Optimal Spectral Wavebands

**Figure 6:**
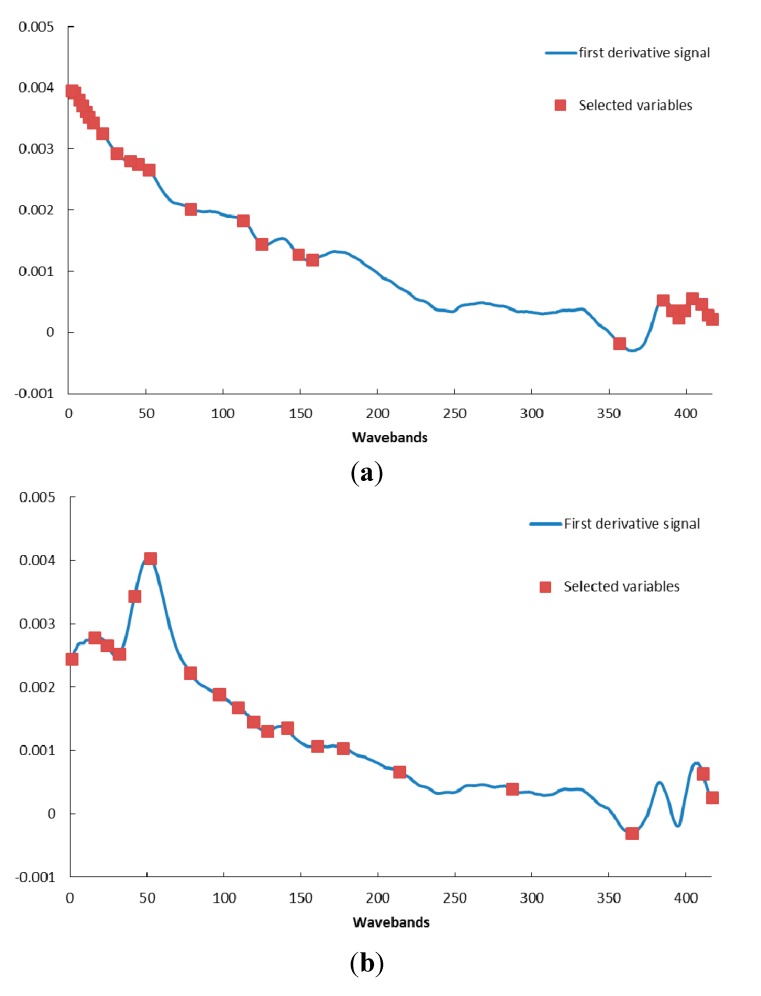
Selected variables using SPA, spectra extracted from germ-up (**a**) and germ down (**b**) images.

As described above, the raw spectral data were preprocessed by SG smoothing and derivation. After this, the optimal wavelength spectra were selected by SPA. SPA was proposed as a novel method to minimize variable collinearity and select the optimal variable [31]. This algorithm started with one wavelength, and then added a new one in each iteration process, and a specified number of wavelengths were selected at the end. The selections of optimal wavebands are shown in Figure 6 and Table 1. The results of wavelength selection are related to the image type from which the spectra information was extracted. The most optimal wavebands of germ-up images concentrated in the regions of lower wavelength (<500 nm) and higher wavelength (800–940 nm), as the most optimal wavebands of germ-down images were located in the region of 500–650 nm. In terms of composition, the germ side contains starch, oil (in the embryo), and other chemical compounds. The leading composition is starch in the endosperm side. Accordingly, the oil- and starch-related bands were reflected in the optimal wavebands, respectively.

**Table 1:** The final selected wavelengths by SPA.

	Selected Wavelengths (nm)
Germ-up images	445.44, 447.85, 451.46, 453.87, 456.29, 458.70, 462.33, 469.59, 480.51, 491.46, 497.56, 506.11, 539.28, 581.40, 596.35, 626.38, 637.68, 891.46, 927.48, 935.20, 940.35, 945.50, 951.93, 959.65, 964.79, 968.65
Germ-down images	444.24, 462.33, 472.01, 481.73, 493.90, 506.11, 538.05, 561.53, 576.42, 588.87, 600.10, 616.35, 641.46, 661.61, 708.45, 801.58, 901.75, 960.94, 968.65

**Figure 7:**
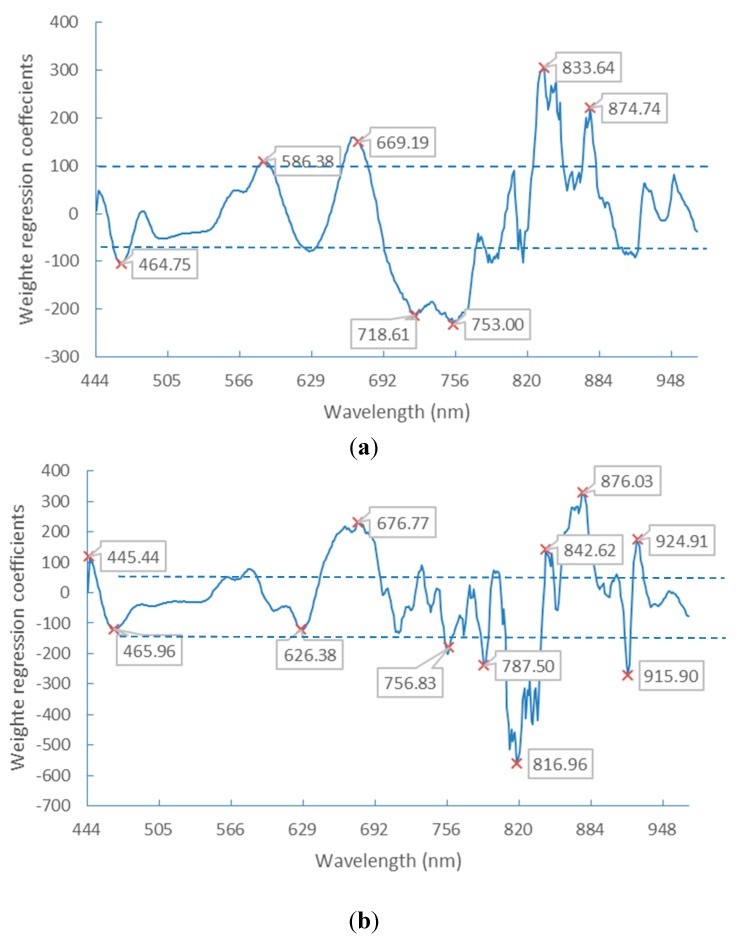
Weighted regression coefficients of the PLS-DA model with selected wavelengths. Spectra extracted from germ-down (**a**) and germ-up (**b**) images.

PLS-DA is another method employed for band selection. It is used to find the fundamental relations between the dependent variables (Y) and the independent variables (X). A latent variable approach is used to model the covariance structures in X and Y spaces. The number of latent variables were chosen based on the minimum root-mean-square error of cross validation (RMSECV) and it was found to be 11 latent variables. The regression coefficients of PLS-DA models, which were obtained from the spectra after SG preprocessing, are show in Figure 7. The wavelengths were selected as the optimal bands according to the highest absolute values of the regression coefficients.

### 3.3. Classification by SVM and PLS-DA

Seed purity is an important standard in seed lot quality. In previous studies, the variety of bulk samples was discussed using the spectra and texture features of bulk samples [26,37], which can’t solve the problem of seed purity testing. In this study, the selected wavelength data by SPA and PLS-DA, as well as the full wavelength of spectral data, morphological, and textual features, were used for individual classification purposes. One hundred kernels of each variety were randomly selected as training sets, and the other samples were used as a test set (50 kernels for each variety sample). SVM and PLS-DA were applied in this research for pattern recognition. In order to improve prediction accuracy and generalization ability, the grid-search and five-fold cross-validation were applied to optimize parameters. The regularization parameter γ and the RBF kernel function parameter σ^2^ were set at the value range from 2^−10^ to 2^10^, respectively. In the PLS-DA model, the number of latent variable was set as 11. The method of SVM is especially suitable to the small dataset and high dimension feature space learning case. Comparing the accuracies of prediction listed in Table 2, the performance of SVM is better than PLS-DA on most types of selected input datasets, except on the dataset selected by PLS-DA.

**Table 2:** The SVM and PLS-DA average classification accuracies (%) of predict set, including both types of images.

Image Type	Classification Method	Full Bands	Image Features	Selected Bands		Features Fusion	
				SPA	PLS-DA	SPA + Image Features	PLS-DA + Image Features
Germ-Up Images	SVM	94.6	86.6	96.2	66.1	98.2	77.4
	PLS-DA	91	65.8	82	83.1	86.4	83.5
Germ-Down Images	SVM	89.2	86.8	95	78.6	96.3	86.1
	PLS-DA	88.4	67.4	86.5	86.8	91.6	86.8

#### 3.3.1. Classification Using Spectral Features

When the raw spectral data was applied in building the SVM classification model, the average accuracy for prediction classification was 94.6% on the germ-up dataset and 89.2% on the germ-down dataset. In the case of optimal wavelength-selected wavebands by SPA, the average classification accuracy was 96.2% on the germ-up dataset and 95.0% on the germ-down dataset. The classification accuracies of full wavelengths spectral data can be rivaled by the optimal wavebands for analysis. In the full-band spectra case, the correlation between adjacent wavebands and high dimensionality might even lead to worse results. Pre-processing the high dimensional data into a lower-dimensional form is a good solution. The method of dimensionality reduction is very important. The results shown in Table 2 illustrate that more satisfactory classification results may be obtained using fewer features extracted by SPA than PLS-DA.

#### 3.3.2. Classification Using Image Features

When morphologic and texture features were used as input for SVM and PLS-DA, SVM produced a better performance and accuracy on the dataset of both sides of the kernel, as shown in Table 2. However, it is not as preferable as the accuracy of full-band spectra. This is due to the shape of maize kernels being not described sufficiently. In the previous studies of rice or wheat, samples have a more regular morphological form than maize kernels and a satisfactory accuracy could be obtained with these simple features [32,33]. However, in the maize case, the larger number of appearance features required more complicated extraction algorithms for satisfactory results. For example, in the research made by Chen *et al*. [2], 17 geometric features, 13 shape features, and 28 color features were extracted and built into the classification model. When morphological characteristics and color were similar among species, it was difficult to classify them by visual methods.

#### 3.3.3. Classification Using both Spectral and Image Features

As analyzed above, employing spectral and image features alone was only good for separating one or several types of maize seeds. Different varieties of maize seeds have varied characteristics revealed in color, composition, and appearance aspects. Spectral data is related to color, moisture, protein, and other components of seeds. According to the discrimination results displayed in Table 2, the classification accuracies using spectral and morphologic fusion data as input were higher than that of using spectral or image features alone. In addition, datasets from both sides of the kernels had the same trend. The identification accuracy of all maize seed varieties reached 98.2% on the germ-up dataset and 96.3% on the germ-down set by SVM. The results were similar to the previous study made by Wang *et al*. [27]. They employed a back propagation neural network and data fusion (optimal wavelength and image data) to classify rice variety discrimination. The best results were based on the spectral and image data fusion.

## 4. Conclusions

A novel method for identifying waxy corn seed purity was proposed and tested in this paper. VIS/NIR HSIs were used in differentiating maize seed varieties. The classification accuracy was related to the features employed for classifier inputs. Although spectral data without any optimized selection to reduce dimension performed well on the dataset, the decreased number of input variables can decrease the training time cost. The satisfactory results were obtained after the raw spectral data were processed by SG smoothing and derivation, and the optimal wavelengths were selected by SPA. Since spectra of maize seeds were affected by variety, growing region, climate, and produce year, *etc.*, employing spectral data alone for analysis is not reasonable for variety classification. The appearance of seeds is the steady genetic characteristic and it is one of the assessments for variety classification. Mathematically, it is difficult to acquire a complete description of the appearance differences between seed cultivars. To solve this problem, both the spectral and image data were chosen as inputs of SVM for classification. Using this method, the classification accuracies reached more than 98.2% and 96.3% for the germ side and the endosperm side, respectively. Finally, we can conclude that VIS/NIR HSI together with spectra and image analysis has the potential to differentiate maize seed varieties and test seed purity effectively.

The kernels used for modeling in this research were produced in the same year. Further research is expected to take the seed sample plant in different regions and years into account to improve the robustness of the classification model. In addition, the spectral range of HSI should be expanded because NIR spectra can explain the difference of chemical constituents between seed cultivars. The morphological and textural descriptions of maize seeds still need to be strengthened. The combination of NIR spectra and more image features will be employed for analysis in our future work.

## References

[1] Payne R. (1986). Variety testing by official AOSA seed laboratories. J. Seed Technol..

[2] Chen X., Xun Y., Li W., Zhang J. (2010). Combining discriminant analysis and neural networks for corn variety identification. Comput. Electron. Agric..

[3] Remund K.M., Dixon D.A., Wright D.L., Holden L.R. (2001). Statistical considerations in seed purity testing for transgenic traits. Seed Sci. Res..

[4] Manickavasagan A., Sathya G., Jayas D.S., White N.D.G. (2008). Wheat class identification using monochrome images. J. Cereal Sci..

[5] Manickavasagan A., Jayas D., White N., Paliwal J. (2010). Wheat class identification using thermal imaging. Food Bioprocess Technol..

[6] Yan X., Wang J., Liu S., Zhang C. (2011). Purity identification of maize seed based on color characteristics. Computer and Computing Technologies in Agriculture IV.

[7] Grillo O., Mattana E., Venora G., Bacchetta G. (2010). Statistical seed classifiers of 10 plant families representative of the Mediterranean vascular flora. Seed Sci. Technol..

[8] Mavi K. (2010). The relationship between seed coat color and seed quality in watermelon Crimson sweet. Hortic. Sci..

[9] Wu D., Feng L., He Y., Bao Y. (2008). Variety identification of Chinese cabbage seeds using visible and near-infrared spectroscopy. Trans. ASABE.

[10] Seregely Z., Deak T., Bisztray G.D. (2004). Distinguishing melon genotypes using NIR spectroscopy. Chemometr. Intell. Lab. Syst..

[11] Delwiche S., Graybosch R.A. (2002). Identification of waxy wheat by near-infrared reflectance spectroscopy. J. Cereal Sci..

[12] Agelet L.E., Hurburgh C.R. (2014). Limitations and Current Applications of Near Infrared Spectroscopy for Single Seed Analysis. Talanta.

[13] Orman B.A., Schumann R.A. (1992). Nondestructive single-kernel oil determination of maize by near-infrared transmission spectroscopy. J. Am. Oil Chem. Soc..

[14] Delwiche S.R. (1995). Single Wheat Kernel Analysis by Near-Infrared Transmittance: Protein-Content. Cereal Chem..

[15] Delwiche S.R., Graybosch R.A., Amand P.S., Bai G. (2011). Starch Waxiness in Hexaploid Wheat (Triticum aestivum L.) by NIR Reflectance Spectroscopy. J. Agric. Food Chem..

[16] Delwiche S.R., Graybosch R.A., Peterson C.J. (1998). Predicting protein composition, biochemical properties, and dough-handling properties of hard red winter wheat flour by near-infrared reflectance. Cereal Chem..

[17] Spielbauer G., Armstrong P., Baier J.W., Allen W.B., Richardson K., Shen B., Settles A.M. (2009). High-throughput near-infrared reflectance spectroscopy for predicting quantitative and qualitative composition phenotypes of individual maize kernels. Cereal Chem..

[18] Cogdill R.P., Hurburgh C.R., Rippke G.R., Bajic S.J., Jones R.W., McClelland J.F., Jensen T.C., Liu J. (2004). Single-kernel maize analysis by near-infrared hyperspectral imaging. Trans. ASAE.

[19] Casasent D., Chen X.W. (2004). Aflatoxin detection in whole corn kernels using hyperspectral methods. Proc. SPIE.

[20] Bauriegel E., Giebel A., Herppich W.B. (2011). Hyperspectral and Chlorophyll Fluorescence Imaging to Analyse the Impact of Fusarium culmorum on the Photosynthetic Integrity of Infected Wheat Ears. Sensors.

[21] Yao H.B., Hruska Z., Kincaid R., Brown R.L., Bhatnagar D., Cleveland T.E. (2013). Detecting maize inoculated with toxigenic and atoxigenic fungal strains with fluorescence hyperspectral imagery. Biosyst. Eng..

[22] Delwiche S.R., Souza E.J., Kim M.S. (2013). Limitations of single kernel near-infrared hyperspectral. imaging of soft wheat for milling quality. Biosyst. Eng..

[23] Williams P., Geladi P., Fox G., Manley M. (2009). Maize kernel hardness classification by near infrared (NIR) hyperspectral imaging and multivariate data analysis. Anal. Chim. Acta.

[24] Mahesh S., Manickavasagan A., Jayas D.S., Paliwal J., White N.D.G. (2008). Feasibility of near-infrared hyperspectral imaging to differentiate Canadian wheat classes. Biosyst. Eng..

[25] Serranti S., Cesare D., Marini F., Bonifazi G. (2013). Classification of oat and groat kernels using NIR hyperspectral imaging. Talanta.

[26] Zhang X.L., Liu F., He Y., Li X.L. (2012). Application of Hyperspectral Imaging and Chemometric Calibrations for Variety Discrimination of Maize Seeds. Sensors.

[27] Wang L., Liu D., Pu H., Sun D.-W., Gao W., Xiong Z. (2015). Use of Hyperspectral Imaging to Discriminate the Variety and Quality of Rice. Food Anal. Methods.

[28] Araújo M.C.U., Saldanha T.C.B., Galvão R.K.H., Yoneyama T., Chame H.C., Visani V. (2001). The successive projections algorithm for variable selection in spectroscopic multicomponent analysis. Chemometr. Intell. Lab. Syst..

[29] Galvão R.K.H., Araujo M.C.U., José G.E., Pontes M.J.C., Silva E.C., Saldanha T.C.B. (2005). A method for calibration and validation subset partitioning. Talanta.

[30] Galvao R.K.H., Araujo M.C.U., Fragoso W.D., Silva E.C., Jose G.E., Soares S.F.C., Paiva H.M. (2008). A variable elimination method to improve the parsimony of MLR models using the successive projections algorithm. Chemometr. Intell. Lab. Syst..

[31] Liu D., Sun D.-W., Zeng X.-A. (2014). Recent advances in wavelength selection techniques for hyperspectral image processing in the food industry. Food Bioprocess Technol..

[32] Paliwal J., Visen N., Jayas D. (2001). Evaluation of neural network architectures for cereal grain classification using morphological features. J. Agric. Eng. Res..

[33] Liu Z.-Y., Cheng F., Ying Y.-B., Rao X.-Q. (2005). Identification of rice seed varieties using neural network. J. Zhejiang Univ. Sci. B.

[34] Pourreza A., Pourreza H., Abbaspour-Fard M.-H., Sadrnia H. (2012). Identification of nine Iranian wheat seed varieties by textural analysis with image processing. Comput. Electron. Agric..

[35] Fernández-Delgado M., Cernadas E., Barro S., Amorim D. (2014). Do we need hundreds of classifiers to solve real world classification problems?. J. Mach. Learn. Res..

[36] Chang C.-C., Lin C.-J. (2011). LIBSVM: A library for support vector machines. ACM Trans. Intell. Syst. Technol..

[37] Choudhary R., Mahesh S., Paliwal J., Jayas D.S. (2009). Identification of wheat classes using wavelet features from near infrared hyperspectral images of bulk samples. Biosyst. Eng..

